# Overexpression of Sterol Carrier Protein 2 in Patients with Hereditary Cholesterol Gallstones

**DOI:** 10.1186/1471-230X-11-10

**Published:** 2011-02-10

**Authors:** YunFeng Cui, ZhongLian Li, ErPeng Zhao, YanFeng Jia, DongHua Li, Ju Zhang, NaiQiang Cui

**Affiliations:** 1 Department of Surgery, Tianjin Nankai Hospital, Nankai Clinical School of Medicine, Tianjin Medical University, 122 Sanwei Road Nankai District, Tianjin 300100, PR China; 2 Institute of Molecular Biology, Nankai University, 94 Weijin Road Nankai District, Tianjin300071, PR China

## Abstract

**Background:**

Lithogenic bile is the major cause of cholesterol gallstone, but its pathogenesis is not well understood. The hypersecretion of biliary cholesterol is believed to be an important cause of lithogenic bile. Sterol Carrier Protein 2 (SCP2) participates in cholesterol trafficking and lipid metabolism in hepatocytes and may play a key role in cholesterol gallstone formation.

**Methods:**

21 cholesterol gallstone genealogies were studied to investigate the expression of SCP2 gene in liver tissue of hereditary and non-hereditary cholesterol gallstone patients as well as non-gallstone patients. The mRNA expression of liver SCP2 in 28 hereditary patients, 30 non-hereditary cholesterol gallstone patients and 32 non-gallstone patients was measured by Reverse Transcription Polymerase Chain Reaction (RT-PCR). The protein expression of liver SCP2 was also detected in all the patients by Western blotting. At the same time, the bile was also analyzed with biochemical techniques and the Cholesterol Saturation Index (CSI) was calculated.

**Results:**

The mRNA and protein expression of SCP2 was significantly increased in cholesterol gallstone patients compared to those of non-gallstone patients. Moreover, SCP2 was expressed at higher levels in hereditary cholesterol gallstone patients than that of non-hereditary cholesterol gallstone patients. There was significant difference observed in CSI between cholesterol gallstone patients and non-gallstone patients, but not in CSI between hereditary and non-hereditary cholesterol gallstone patients.

**Conclusions:**

SCP2 was overexpressed in hereditary cholesterol gallstone patients compared to non-hereditary cholesterol gallstone patients. This finding indicated that SCP2 might be one of the genetic factors contributing to cholesterol gallstone formation, which was always accompanied by the increase of bile lithogenicity.

## Background

Hypersecretion of cholesterol in bile, leading to the formation of lithogenic bile, is believed to be the major cause of cholesterol gallstones [1]. Sterol carrier protein 2 (SCP2), also called nonspecific lipid transfer protein, is a 13.2 KD base protein and exists in peroxisome, mitochondria, endoplasmic reticulum and cytoplasm [2-4]. As a moderating factor of cholesterol metabolism, it is involved in the biosynthesis of cholesterol [5-7] and the transformation of cholesterol to bile acid [8,9], cholesteryl ester [10] and sterols [11]. As a transporting tool, on the other hand, this protein participates in the transportation of cholesterol inside the cell and through the cytoplasm membrane [12,13] as well as the rapid transportation of the newly synthesized cholesterol from endoplasmic reticulum into the bile without the intervention of cytomicrotubule system and Golgi bodies [14]. Hence, hypersecretion of biliary cholesterol with the formation of lithogenic bile may explain the formation mechanism of the cholesterol stones in the gallbladder.

We have found some patients with gallstone to also have familial backgrounds of the same condition. In such cases, individuals were more susceptible to cholesterol stone formation than those individuals lacking this familial background. Therefore, in this study, we have enrolled patients with a familial background in order to determine if differences in SCP2 expression levels and the bile CSI between hereditary and non-hereditary cholesterol gallstone patients exist.

## Methods

We have accumulated 21 cholesterol gallstone families and undertook a study to investigate the expression of SCP2 in liver tissue of hereditary and non-hereditary cholesterol gallstone patients as well as non-gallstone patients by using reverse transcription-polymerase chain reaction and Western Blotting. The bile was also analyzed by means of biochemical techniques and Cholesterol Saturation Index (CSI) was calculated at the same time.

### Case Selections

#### Hereditary cholesterol gallstone group

Patients with cholesterol stones in the gallbladder, cholesterol contents >50% and with a familial background. The criterion of familial background is such that there are no less than two patients who are in different generations of consanguinity in one family.

#### Non-hereditary cholesterol gallstone group

Patients with cholesterol stones in the gallbladder, cholesterol contents >50%, but without a familial background.

#### Control group

Patients with primary intrahepatic cholangiolithiasis and cholesterol contents <20%; patients with peptic ulcer, cancer of the stomach or of the colon in whom no gallstones are verified by ultrasound diagnosis.

#### Exclusion criteria

Patients with diabetes or with other endocrine metabolism disorders; obese individuals; and patients with other diseases of the liver and the gallbladder.

All patients were admitted to and had operations at Tianjin Nankai Hospital between August 2003 and August 2008. Hereditary gallstone group consisted of 28 patients with cholesterol stones in the gallbladder, 15 females and 13 males, aging between 25-80 years. The stones appeared yellow or light yellow in color, with smooth or nodular surfaces. Non-hereditary cholesterol gallstone group consisted of 30 patients with cholesterol stones in the gallbladder, 16 females and 14 males, aging between 20-80 years old. The control group consisted of 32 patients, including 16 females and 16 males, aging between 15-78 years old. Of the 32 patients in the control group, 8 patients suffered from primary hepatocholedocolithiasis with brown stones which were pigment stones, 5 patients were diagnosed with gastric cancer, 11 patients with non-cholesterol polyp in the gallbladder, and 8 with pancreatic cancer. There were no significant differences between the three groups when compared by the age and the body mass index (P > 0.05).

### Bile and Liver Sampling

Patients fasted for 12 hours before the operation and general anaesthesia was administered. A liver specimen of 50 mg was obtained and bile collected from gallbladder. Bile was kept at -20°C while liver tissues were stored at -70°C until it was processed. Biopsies were permitted by Hospital Ethics Committee and the consents obtained from the patients and family members.

### Identification of Cholesterol Stones

Stones obtained during the operation were rinsed with water and put in the dryer until a constant weight was obtained. Then the stones were ground and dried naturally for 12 hours. A sample of 10 mg gallstone powder was weighed and dissolved in 5 ml anhydrous alcohol, stirred for 3 minutes, and centrifuged at 3000 rpm for 5 minutes. The supernatant was collected for further analysis. Another 10 mg sample was accurately weighed, dissolved in 5 ml chloroform, stirred for 3 minutes, centrifuged at 3000 rpm for 5 minutes, and the supernatant was collected for analysis. The cholesterol and calcium bilirubinate were measured by enzyme method. The ratio of cholesterol content to calcium bilirubinate content was calculated and used for sample classification. Samples with a ratio >0.5 were classified as cholesterol stones, and those with a ratio <0.5 as pigment stones [15].

### Reverse-Transcription Polymerase Chain Reaction

Total RNA was prepared from liver tissue by using Trizol method and 1 μg RNA was reverse-transcribed by using random primer hexamers. Reverse-transcription polymerase chain reaction (RT-PCR) was performed by using primers based on the human SCP2 cDNA sequence [M55421.1] from Genebank. For SCP2, upper Primer is 5'-ATGGGTTTTCCGGAAGCCGCCAGTT, lower primer is 5'-TCAGAGCTTA GCGTTGCCTGGCTG, and the PCR fragment was a product of 432 bp. For β_2_-Microglobulin, upper Primer is 5'-ATGCCTGCCGTGTGAACCATGT, lower primer is 5'-AGAGCTACCTGTGGAGCAACCT, and the PCR fragment was a product of 285 bp. SCP2 and β_2_-Microglobulin genes were amplified at the same time, under the same conditions. PCR was carried out as follows: one cycle of 94°C for 3 min, and 22 cycles of 94°C for 1 min, 58°C for 1 min, 72°C for 1 min, and one cycle of 72°C for 3 min. After electrophoresis, PCR products were quantified by the Gene Genius Gel Documentation and Analysis System, and SCP2 expression level was normalized to β_2_-Microglobulin level.

### Western Blot

The tissues were lysed with the lysis buffer (50 mM Tris PH7.4, 150 mM NaCl, 0.5% Triton X-100, 1 mM EDTA, 1 mM PMSF). Total protein was separated by 12% sodium dodecyl sulfate polyacrylamide gel electrophoresis and electrophoretically transferred onto polyvinylidene difuoride membrane (Millipore, Bedford, MA, USA). After blocking non-specific binding sites with 5% non-fat milk, the membrane was incubated with primary antibodies for 1 h at room temperature. After washing, the blots were incubated with horseradish peroxidase-conjugated anti-mouse or anti-rabbit IgG, and immunoreactive bands were visualized with Western Blotting Luminol Reagent kit (Santa Cruz Biotechnology Inc). Densitometric analysis was used to select the contour of the band, subtract the background and calculate the density. The primary antibodies used in this experiment were as follows: anti-SCP2 antibody (kindly provided by Dr Schroeder, Texas A&M University, 1:1000 dilution) and anti-GAPDH antibody (Santa Cruz Biotechnology, 1:2000 dilution).

### Bile Biochemical Analyses

The concentrations of biliary cholesterol, phospholipids, and total bile acids were measured using enzymatic kits (Zhongsheng Bioengineering High-Tech Co.).

### Data Analysis

All data were expressed as mean ± standard deviation. Difference between control and experimental groups were evaluated by one-way analysis of variance (ANOVA). P < 0.05 was considered statistically significant (a = 0.05).

## Results

### The mRNA and protein expression of SCP2 in hereditary gallstone group, non-hereditary gallstone group and control group

**Figure 1:**
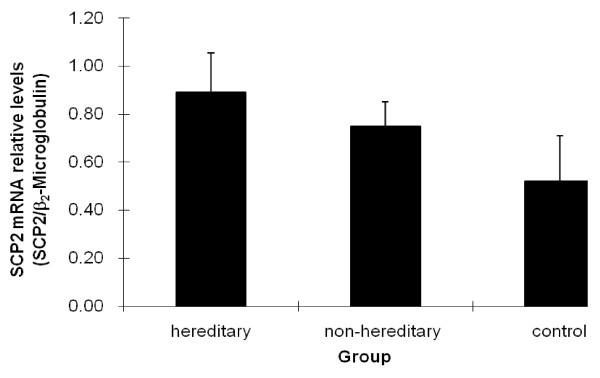
**The mRNA levels of SCP2 in hereditary gallstone group, non-hereditary gallstone group and control group**. SCP2 mRNA expression was assessed by semi quantitative RT-PCR and shown as fold change after normalized to β_2_-Microglobulin levels. SCP2 mRNA was expressed at higher levels in hereditary cholesterol gallstone patients than in non-hereditary cholesterol gallstone patients, P < 0.05. The mRNA expression of SCP2 in hereditary, and non-hereditary cholesterol gallstone patients were 0.8908 ± 0.1649, 0.7503 ± 0.1004 respectively, which were both higher than that in control group,0.5205 ± 0.1900, P < 0.05. The data was analyzed by one way analysis of variance, and F = 11.089, P < 0.05.

We detected mRNA and protein expression of SCP2, the main cholesterol transporter and mediator in hepatocytes. As shown in Figure 1, SCP2 mRNA was expressed at higher levels in hereditary cholesterol gallstone patients compared to those of non-hereditary cholesterol gallstone patients. The expression of SCP2 mRNA in hereditary as well as non-hereditary cholesterol gallstone patients was significantly higher than that of the control group. Three groups were compared by one way analysis of variance, and F = 11.089, P < 0.05.

**Figure 2:**
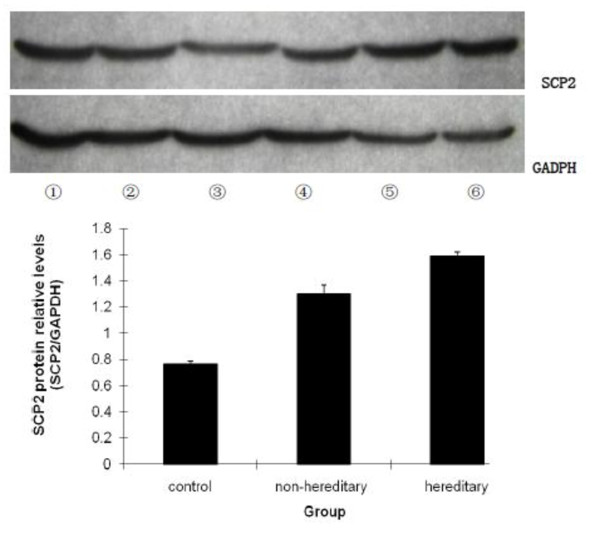
**The protein levels of SCP2 in hereditary gallstone group, non-hereditary gallstone group and control group**. All samples were analyzed by Western blot using anti-SCP2 antibody, and GAPDH was used as the internal control. Results were shown as SCP2/GAPDH ratio in arbitrary densitometric units representing SCP2 protein relative levels. Lane ① and ② are from control group, Lane ③ and ④ from non-hereditary group, and Lane ⑤ and ⑥ from hereditary group. SCP2 protein was expressed at higher level in hereditary cholesterol gallstone patients than that in non-hereditary cholesterol gallstone patients, and the difference was statistically significant, P < 0.05. SCP2/GAPDH ratios in hereditary, and non-hereditary cholesterol gallstone patients were 1.5898 ± 0.0316, 1.3032 ± 0.0664 respectively, which were both higher than that in control group,0.7637 ± 0.0258, P < 0.05.

We analyzed the expression of SCP2 in all samples by Western blot and GAPDH was used as the internal control. Results were shown in Figure 2 as SCP2/GAPDH ratio. Consistent with the expression level of SCP2 mRNA, SCP2 protein was expressed at higher levels in hereditary cholesterol gallstone patients than in non-hereditary cholesterol gallstone patients, P < 0.05. SCP2 protein relative levels in hereditary and non-hereditary cholesterol gallstone patients were 1.5898 ± 0.0316 and 1.3032 ± 0.0664, respectively, which were both higher than that in control group, 0.7637 ± 0.0258, P < 0.05. Three groups were compared by one way analysis of variance, F = 1084.98, P < 0.05.

### Changes of bile components and lithogenicity between hereditary, non-hereditary cholesterol gallstone patients and control groups

**Figure 3:**
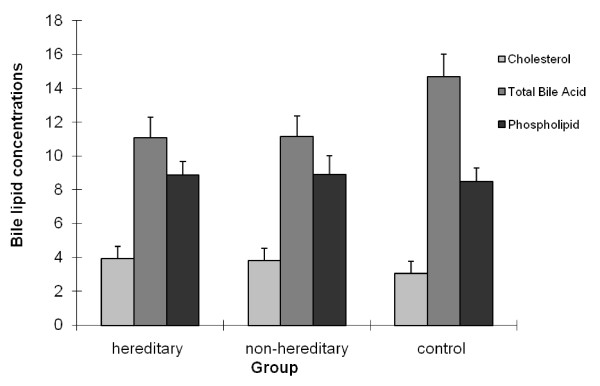
**Changes of gallbladder bile Cholesterol, Total Bile Acids and Phospholipids in hereditary, non-hereditary cholesterol gallstone patients and control group**. Bile cholesterol in hereditary, and non-hereditary cholesterol gallstone patients were both higher than that in control group, P < 0.05. But there was no significant difference in bile cholesterol level between hereditary and non-hereditary cholesterol gallstone patients. Three groups were compared by one way analysis of variance, F = 84.230, P < 0.05. Total bile acids level in hereditary and non-hereditary cholesterol gallstone patients were both lower than that in control group, P < 0.05. There was no significant difference in Total bile acids level between hereditary and non-hereditary cholesterol gallstone patients. Three groups were also analyzed by one way analysis of variance, F = 17.382, P < 0.05. There were no significant differences in Phospholipids level between three groups. Three groups were also compared by one way analysis of variance, F = 0.492, P > 0.05.

The concentrations of cholesterol, phospholipids and bile acids in gallbladder bile were obtained and the CSI was calculated using the Carey table. As shown in Table 1 and Figure 3, we have found cholesterol in hereditary and non-hereditary cholesterol gallstone patients to be higher than that in control group. Total bile acids in hereditary and non-hereditary cholesterol gallstone patients were both lower than that in control group. Additionally, no significant difference in Phospholipids between the three groups was observed.

**Table 1:** The concentrations of Cholesterol, Total Bile Acids and Phospholipids in gallbladder bile

group	**Cholesterol **^**a **^**(mmol/L)**	**Total Bile Acids **^**b**^**(mmol/L)**	**Phospholipids **^**c **^**(mmol/L)**	**CSI **^**d**^
control group	3.0620 ± 0.7053	14.6700 ± 1.3316	8.4880 ± 0.8190	0.7342 ± 0.1550
non-hereditary group	3.8333 ± 0.7217**	11.1667 ± 1.2010**	8.8933 ± 1.1162**	1.5190 ± 0.2715**
hereditary group	3.9200 ± 0.7229*	11.0900 ± 1.2038*	8.8600 ± 0.8050*	1.5810 ± 0.2400*

a*compared with control group *P *< 0.05, compared with non-hereditary group *P *> 0.05; **compared with control group *P *< 0.05

b*compared with control group *P *< 0.05, compared with non-hereditary group *P *> 0.05; **compared with control group *P *< 0.05

c*compared with control group *P *> 0.05, compared with non-hereditary group *P *> 0.05; **compared with control group *P *> 0.05

d*compared with control group *P *< 0.05, compared with non-hereditary group *P *> 0.05; **compared with control group *P *< 0.05

CSIs in hereditary and non-hereditary cholesterol gallstone patients were both higher than that in control group, P < 0.05; however, no significant difference was observed in CSIs between hereditary and non-hereditary cholesterol gallstone patients. Three groups were compared by one way analysis of variance, F = 49.166, P < 0.05.

## Discussion

Cholesterol gallstone formation is a complicated process involving a variety of factors. The abnormal metabolism of liver cholesterol and supersaturation of bile cholesterol are the major causes of stone formation. In recent years, some scholars have finished a series of epidemiological studies in high-risk populations about cholelithiasis [16-18] and have made some interesting findings. The gallstone disease is a genetic disease involving multiple genes and having the genetic characteristic of autosomal dominant delay. Gallstone formation is caused by common genetic factors along with multiple environmental factors. Interactions between related genes and environmental factors play an important role in the pathogenesis of gallstone formation.

Ito, et al [19]conducted an analysis of three regulatory enzymes in the cholesterol metabolism, as well as cholesterol levels and sterol carrier protein 2/nonspecific lipid transfer protein (SCP2/nsLTP) levels by using liver biopsy samples of Japanese patients who had cholesterol gallstones and underwent cholecystectomy. His results suggest that intracellular cholesterol transport is enhanced in patients with cholesterol gallstones. Animal experiments confirmed SCP2 was an essential factor in the transportation of newly synthesized cholesterol into bile; rapidly transfering cholesterol from endoplasmic reticulum directly into the bile without the involvement of the cellular microtubule system and the Golgi [20]. Fuchs, et al [11,21] observed the phenomenon in the stone susceptible mice where SCP2 protein and SCP2 mRNA arose at the same time and thought that the transcriptional upregulation of SCP2 led to the higher level of SCP2 in liver cell, then increasing bile cholesterol and promoting the gallstone formation. Further studies on susceptible mice found that Lith 1 gene might result in overexpression of SCP2 mRNA during the formation of gallstones.

In our previous studies [22,23], we have found SCP2 mRNA to be overexpressed in cholesterol gallstone patients compared to the control group (P < 0.05), which was consistent with other studies [11,19,21]. Interestingly, we have also found that some patients with gallstone gathered in some families and the individuals with a familial background were more susceptible to form cholesterol stones than those without a familial background. Based on this initial finding, heredity could be another factor contributing to cholesterol gallstone formation. To elucidate the possibility, we have enrolled into our research patients with a familial background and increased the number of cases in order to determine whether or not the differences in SCP2 expression and the bile CSI can be observed between hereditary and non-hereditary cholesterol gallstone patients.

We have found mRNA and protein expression of SCP2 in hereditary cholesterol gallstone group to be significantly higher than that of non-hereditary cholesterol gallstone group, (P < 0.05). In addition, we have also found mRNA and protein expression of SCP2 to be higher in non-hereditary cholesterol gallstone group than that of control group. Compared with the study of Ito et al, we have observed higher expression of SCP2 in hereditary cholesterol gallstone group than that of non-hereditary cholesterol gallstone group. The different levels of SCP2 expression between hereditary and non-hereditary cholesterol gallstone groups indicatethat SCP2 as one of the genetic factors in the formation of cholesterol gallstones. An overexpression of SCP2 in the liver may lead to higher concentration of cholesterol in the bile, causing bile lithogenicity to exceed the normal borderline, which could cause the cholesterol crystals to separate out and form the initial stones in the bile. The increased SCP2 levels can also accelerate the transportation of cholesterol inside hepatocytes and the excretion across the membrane of liver cells. There are also some other possibilities that SCP2, as a moderator, may regulate the metabolic process of cholesterol, such as HMGCR, 7alpha-hydroxylase and ACAT. We have found lithogenicity of bile in cholesterol gallstone patients to be higher than that of control group, but there was no significance difference in the lithogenicity of bile between hereditary and non-hereditary cholesterol gallstone groups. So the formation of gallstones was always accompanied by the higher lithogenicity of bile.

Beyond the relationship between SCP2 expression and cholesterol gallstone formation, the underlying molecular mechanisms are still unclear, especially those involved in the relationship between SCP2 and familial backgrounds.

## Conclusions

SCP2 may be a genetic factor influencing the susceptibility to the formation of gallstones under the same life environment, and overexpression of SCP2 could be one of the important causes of cholesterol gallstones. The formation of gallstones is always accompanied by the increase in bile lithogenicity, which is not heredity-dependent.

## Competing interests

The authors declare that they have no competing interests.

## Authors' contributions

YFC performed the majority of experiments; DHL and YFJ took part in some experiments; NQC and JZ involved in critical reading and helpful discussion of the manuscript; ZLL and EPZ provided the collections of human liver and bile samples; YFC and NQC designed the study and wrote the manuscript. All authors read and approved the final manuscript.

## Pre-publication history

The pre-publication history for this paper can be accessed here:

http://www.biomedcentral.com/1471-230X/11/10/prepub
